# An Alternative, High Throughput Method to Identify *Csd* Alleles of the Honey Bee

**DOI:** 10.3390/insects11080483

**Published:** 2020-07-30

**Authors:** Éva Kolics, Tamás Parrag, Ferenc Házi, Kinga Szepesi, Botond Heltai, Kinga Mátyás, Barbara Kutasy, Eszter Virág, János Taller, László Orbán, Balázs Kolics

**Affiliations:** 1 Department of Biotechnology, Georgikon Faculty, University of Pannonia, H-8360 Keszthely, Hungary; kolicseva@gmail.com (É.K.); parrag.tamas@georgikon.hu (T.P.); szepesikinga66@gmail.com (K.S.); heltaib2@gmail.com (B.H.); petrovicsnemkk@gmail.com (K.M.); kutasybarbara@gmail.com (B.K.); virag@georgikon.hu (E.V.); taller@georgikon.hu (J.T.); 2 Kolics Apiaries, H-8710 Balatonszentgyörgy/H-7586 Bolhó, Hungary; 3 Private Beekeeper, H-5520 Szeghalom, Hungary; szitor@gmail.com; 4 Frontline Fish Genomics Research Group, Department of Animal Sciences, Georgikon Faculty, University of Pannonia, H-8360 Keszthely, Hungary; orban.laszlo@georgikon.hu

**Keywords:** *Apis mellifera*, hypervariable region, alleles of *csd* gene, molecular markers, queen breeding

## Abstract

Applying instrumental insemination in closely related honey bee colonies often leads to frequent lethality of offspring causing colony collapse. This is due to the peculiarities of honey bee reproductive biology, where the *complementary sex determination* (*csd*) gene drives sex determination within a haplodiploid system. Diploid drones containing homozygous genotypes are lethal. Tracking of *csd* alleles using molecular markers prevents this unwanted event in closed breeding programs. Our approach described here is based on high throughput sequencing (HTS) that provides more data than traditional molecular techniques and is capable of analysing sources containing multiple alleles, including diploid individuals as the bee queen. The approach combines HTS technique and clipping wings as a minimally invasive method to detect the complementary sex determiner (*csd*) alleles directly from honey bee queens. Furthermore, it might also be suitable for screening alleles of honey harvested from hives of a closed breeding facility. Data on alleles of the *csd* gene from different honey bee subspecies are provided. It might contribute to future databases that could potentially be used to track the origin of honey. With the help of tracking *csd* alleles, more focused crossings will be possible, which could in turn accelerate honey bee breeding programmes targeting increase tolerance against varroosis as well.

## 1. Introduction

More than fifty years before the discovery of sex chromosomes [1], it was reported that unfertilized eggs of the honey bee (*Apis mellifera* L., 1758) develop into haploid drones, whereas fertilized ones develop into diploid females [2]. Haplodiploidy can be observed in about 20% of all animal species, comprising the entire insect order of Hymenoptera [3]. Unfortunately, the genetic basis of their sex determination (SD) appears to be diverse and poorly understood [4]. First, it was discovered in a wasp *Bracon hebetor* that diploid males hatched from fertilized eggs are highly unviable [5]. Later, the same phenomenon was reported from the honey bee [6]. Based on these findings, it was suggested that the *complementary sex determiner (csd)* gene represents the primary signal that directs sexual development [7,8,9]. The product of *csd* is encoded by an autosomal locus within the SD cascade. This SD mechanism is called single-locus or multiple-locus complementary sex determination (sl-CSD or ml-CSD) where homozygosity of the *csd* alleles results in males, irrespective of their ploidy level [10].

Homozygous diploid honey bee males are unviable. During larval stage, they are eaten by the workers upon their detection. This trait in the colony is unfavourable due to the reduction in the number of worker bees resulting in inappropriate colony growth and production loss. Honey bee queens naturally tend to avoid mating in the vicinity of their hive [6]. Drone congregation areas help to establish a batch of different dronal alleles for the nuptial flight. The mating of a queen with multiple drones also increases the diversity of alleles in the hive, which is a superorganism. Labour-intensive closed breeding programmes (e.g., for *Varroa* tolerance) lack these evolutionary assurances; diploid drones may appear quickly, and inbreeding remains a big threat without knowing the *csd* alleles, especially in the case of single drone insemination [11].

According to morphometric and genetic data, *A. mellifera* can be divided into four evolutionary clades [12,13,14]. These subspecies bear economic importance, out of which *A. m. ligustica* and *A. m. carnica* are kept worldwide commercially [15]. Several alleles of various honey bee subspecies were described based on the hypervariable (HVR) region [11,16,17,18]. This particular region of the *csd* gene was selected for the typing of *csd* alleles in breeding programmes. The evolutionary rate, on average, is about 2.4 faster within the HVR relative to microsatellites. Even within a short evolutionary time, this fact confirms the contribution of the HVR to csd-allelic variability [19]. The genes of honey bees tend to show high levels of polymorphism [16,20]. According to Wang, et al. [21], *A. mellifera* subspecies *A. m. carnica* and *A. m. ligustica* also show a high level of polymorphism in *csd* genes.

A cost-effective technology based on High Resolution Melting (HRM) analysis has been in commercial use in a queen breeder company. In their approach, *csd* alleles have differences in the amino acid sequence at the HVR region of *csd* [11]. However, this method may be restricted in practice for the specific apiary analysed, as the appearance of new alleles necessitates the assignment of additional sequences to relevant new HRM curves, and same-sense mutations may result in different ones. In the current approach, *csd* alleles of a queen are deduced through the determination of *csd* sequences of her descendant males. Furthermore, this method is time-consuming due to the delayed and restricted appearance of males. Alternatively, high throughput sequencing of samples originated from the queen itself would precede a labour intensive cloning process [16].

Diagnostic methods used in honey analysis for food control imply DNA-based techniques, focusing on the botanical origin of the honey [22,23] and the identification of its entomological origin via molecular markers [24,25,26,27,28]. According to our knowledge, this approach has not been used for tracking the genetic composition of a mating yard thus far.

In order to determine the *csd* gene variations for obtaining pre-insemination information on queens and avoiding the appearance of homozygous diploid males during the breeding process, we applied a new method. Our technique targets the HVR region of *csd* gene and is compatible with high-throughput sequencing technologies. In the case of honey, the aim was to test whether it is possible to gain information on *csd* alleles from an extremely high complex mixed sample in such an unfavourable environment.

## 2. Material and Methods

### 2.1. DNA Extraction from Bees

Bee samples were collected from private apiaries as detailed in Table 1. Non-lethal DNA extraction was carried out in the case of queen and worker bees using their clipped wings [29]. In the case of the queen bees, the sampling was made considering the aim of the examination. For insemination purposes, the newly hatched queen should be used, while tracking of the breeding lines during the examination can be conducted upon the colony selected for breeding.

DNA was extracted from cut hind wings of 4 workers and 4 queen bees as well as from 4 drone pupae (some in technical iteration). Samples were stored in ethanol at −20 °C before use. For the DNA extraction, we used a small amount of tissue from the clipped wings of the bees or homogenized tissues from pupae. All DNA extractions were carried out using Qiagen DNeasy Tissue Kit (QIAGEN GmbH, Hilden, Germany) according to the instructions of the manufacturer. The yield and the quality of the extracts were determined by spectrophotometry using NanoDrop™ 2000/2000c Spectrophotometer (Thermo Scientific™, Waltham, MA, USA). Yields of DNA ranged between 3.14 and 9.13 ng/µL.

### 2.2. DNA Extraction from Honey

Honey samples were collected from private apiaries and groceries (Table 1). DNA from honey was extracted the using the same kit for bees, with slight modifications implemented as follows. Honey was diluted 1:10 in water and pelleted in 30 mL tubes at 15,000 rpm. The pellet was decanted and incubated in Buffer ATL with 10 µL Proteinase K (20 µg/µL) after a homogenisation in a bead mill for 5 min at 20 rpm using beads of 3 mm diameter.

### 2.3. Sequencing of csd Alleles

The HVR region of the *csd* gene was amplified using primers HVR-F (5′-AGTACCTAAAATAATTTCATCTTTATC-3′) and HVR-R (5′-TGCCAAAATCTTGGTATTTGTTCTTG-3′) flanked by Illumina Nextera adaptor sequences (5′-TCGTCGGCAGCGTCAGATGTGTATAAGAGACAG-3′) and 5′-GTCTCGTGGGCTCGGAGATGTGTATAAGAGACAG-3′) for Illumina MiSeq sequencing. For each honey bee sample, PCR was performed as follows: an initial pre-amplification denaturation period at 98 °C for 3 min was followed by 37 cycles at 98 °C for 10 s, 61 °C for 20 s, and 72 °C for 20 s and ended with a final extension at 72 °C for 2 min. Amplicon sizes varied according to the actual size of the certain allele ranging between 284 bp to 317 bp for the total size of the *csd* locus; whilst remaining restricted to the HVR region within, it varied between 87 to 144 bp.

Locus-specific PCR products were further purified using 1.0 volume KAPA PureBeads (F. Hoffmann-La Roche, Switzerland) according to the manufacturer’s protocols. The concentration of eluted DNA was measured using a Qubit 3.0 Fluorometer with Qubit dsDNA HS Assay Kit (Thermo Fisher Scientific). Index PCR reactions (20 µL each) were set up by using 20 ng of purified template in 6 µL, 2-2 µL Nextera XT Index kit v2 Primers (N7xx & S5xx) (Illumina, Inc. San Diego, CA, USA), and 10 µL of 2xKAPA Hifi Hot Start Ready Mix (F. Hoffmann-La Roche, Switzerland). PCR cycling parameters for index PCRs were as follows: initial denaturation at 95 °C for 3 min; 8 cycles at 95 °C for 30 s, 55 °C for 30 s, 72 °C for 30 s; final extension at 72 °C for 5 min. PCR products were purified using 1.0 volume KAPA PureBeads and eluted in 20 µL of 10 mM Tris-HCl pH 8. The product libraries were quantified and qualified by using High Sensitivity D1000 ScreenTape on TapeStation 2200 instrument (Agilent Technologies, Santa Clara, CA, USA). Equimolar concentrations of libraries were pooled, diluted to 4 nM, and combined with other sample pools to gain the desired sequencing depth.

**Figure 1:**
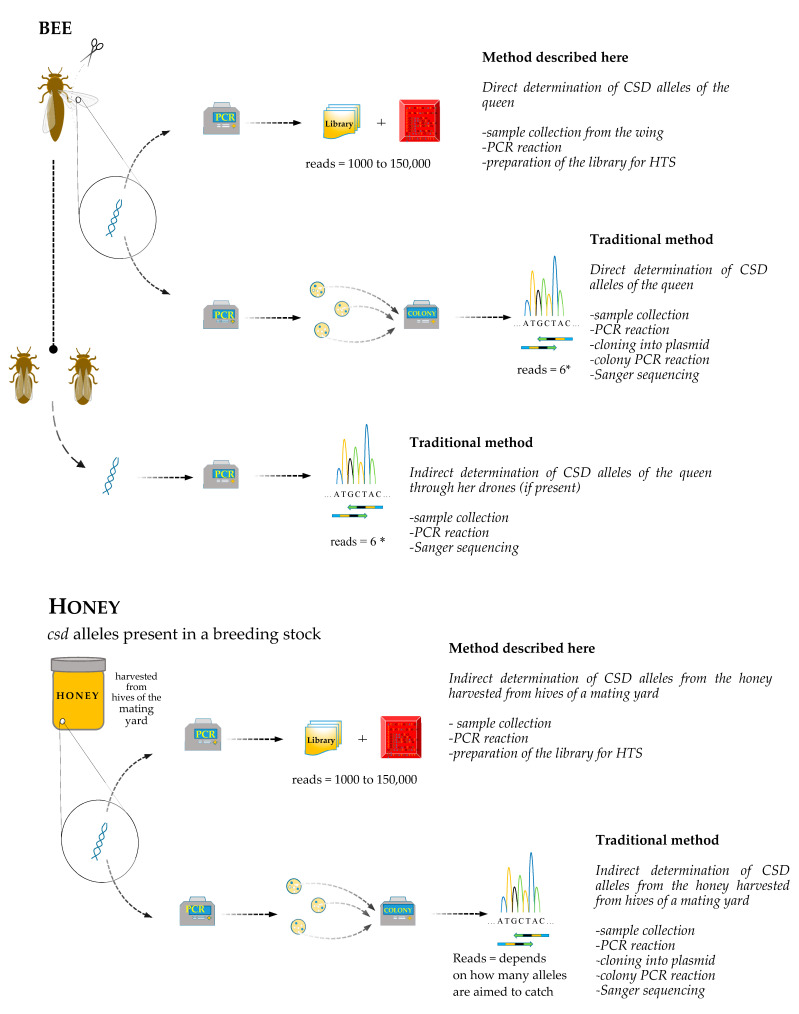
Comparison of the two methods shows that traditional methods provides alternative only when drones are available with a compromise of more DNA extraction and PCR. However, if queen is directly targeted, it leads to extensive molecular cloning and sequencing. The advantage of the novel approach is disputable when investigating bulk samples.* In order to successfully catch both alleles, 6 sequencing is optimal.

Sequencing was carried out using Illumina MiSeq platform and 600-cycle Reagent Kit v3 (Illumina Inc., San Diego, CA, USA). Samples were demultiplexed and adapter-trimmed by using MiSeq Control Software. Schematic figure shows an overview and comparison of present methods (Figure 1).

### 2.4. Bioinformatic Analysis

Raw sequences were first filtered for fragment length of a minimum of 50 base pairs and quality scores of ≥Q30 using Trimmomatic v.0.36 software [30] and were subsequently merged using Pear v.0.9.5 software [31]. Finally, sequences were clustered using Usearch v.11.0.667 software [32]. Each script was applied according to default settings. Sequences were aligned against reference honey bee *csd* sequences from NCBI GenBank database (ID: KF741286.1). Only those sequences that contained the entire forward and reverse PCR primers were considered. Then, identical sequences were clustered using search scripts. Sequences for each PCR sample were then translated into proteins. Alleles were regarded as sequences resulting in different amino acid sequence in the HVR region according to Hyink, Laas and Dearden [11]. Threshold was set above the abundance to a minimum of 1000 identical copies of the same sequence.

## 3. Results

### 3.1. Individual csd Alleles Might Be Genotyped from Samples Containing Multiple Alleles

We investigated the applicability of sex determination *(csd)* region genotypes of honey bee for rapid genetic assessment of hives through the testing of queen bees or honey by high throughput sequencing. Altogether, 311,973 raw sequences were gained from 12 bee individuals, out of which seven samples—three queen bees (Hq1, Hq2, Hq3), two workers (Cw3, Hw1), and two drones (Hd1, Hd3)—were used as two technical replicates per each sample labelled as: Hq1.1, Hq1.2, Hq2.1, Hq2.2, Hq3.1, Hq3.2, Cw3.1, Cw3.2, Hw1.1, Hw1.2 as well as Hd1.1, 1.2. and Hd3.1, Hd3.2. A subsequent filtering for quality control—dropping unpaired reads and filtering for minimum length of 120 bases using Trimmomatic scripts as well as filtering for unassembled sequences—left 299,370 filtered sequences to be aligned against a reference *csd* allele sequence. After alignment and subsequent translations to the relevant open reading frame, 255,339 clean reads were analysed. The iteration Hd1.3 was handled separately as a control, starting from 150,801 reads and filtered to 149,407, leaving 139,098 sequences for the analysis.

The primers successfully amplified the HVR region, yielding the same allele in the iterations (see upper indexed samples in Table 1 and Table 2). However, there were differences in the abundances and the coverage that may have been the consequence of differences in amplification efficacy. On the other hand, the number of the expected alleles is known in common cases where awareness of *csd* alleles is required: two for the diploid queen and one for each haploid drone. The number and the abundance of discarded alleles of queen bees and some drone samples can be found in Table S1.

The *csd* allele of a drone individual (Hd1.3) isolated from hive 445/23 was sequenced alone as well as in a single lane as a positive control. This sample yielded 139,098 reads that translated into a single amino acid sequence covering 93.1% of the total sequences of the dataset of this lane. 

From honey, we sequenced eight samples. In total, 1,975,885 sequences were gained and filtered to 1,864,415 clean reads using the same pipeline as in the case of the bees. We needed ten times more data from honey than those from bees, as the samples contained *csd* alleles from numerous hives (60–400 in the case of samples collected from known apiaries). The Hw1.1_SA1 *csd* allele was scattered through the dataset and found in the drone of the hive 445/23 (Hd1.1, Hd1.2, Table 2), in its female hive mate (Hw1.1), in the honey from this hive, and in a honey mix that contained honey from this colony.

### 3.2. Novel csd Alleles Were Uncovered Using High Throughput Sequencing

Altogether, seven novel *csd* alleles for *Apis mellifera carnica/ligustica/caucasica* were identified and detailed in Table 3 and Table S2. New alleles were found in two *Apis ligustica* worker bee samples and one honey sample. Eight further honey samples included five new alleles, corresponding respectively to *Apis mellifera carnica* and *Apis mellifera caucasica* (Table 3). The result of our work confirmed that the HVR region contains several different alleles—in total, a number of 25 alleles (Figure S1). Only alleles that were sequenced from at least two different samples were considered to be new. These sequences with adherent data were deposited in NCBI under the Accession MK241931.1-MK241937.1.

## 4. Discussion

### 4.1. A High-Throughput Sequencing Technique is Provided for Detection of the csd Alleles of a Breeding Queen

In the current practice of genotyping of honey bee queens for the *csd* locus, nucleotide sequences of their *csd* alleles are determined indirectly through genotyping of their male offspring, if available. This has obvious restrictions for queen breeding practice, since drones are present only during certain parts of the year. Indeed, they may be even absent during the whole first year since young queens typically produce few or no drone brood. This is especially true for queens from breeding programmes, where swarming drive is one of the most important selection criteria. Sequencing the *csd* alleles gives opportunity to select the most proper queen for insemination. Until recently, alleles have mainly been genotyped by Sanger sequencing [16,21] or, most recently, by an HRM-based technique [11]. However, *csd* allele typing directly from the queen, which would be able to provide sequence information, was lacking prior to this study. The method described here allows for sequence-based identification of *csd* alleles from the wing of a queen bee, establishing the first HTS-based genotyping for this locus and giving the opportunity for breeders to monitor their breeding lines. We chose this sampling method as the wings are the only part of her body that can be clipped without exposing her to be recognised by her colony as fatally handicapped, resulting in unwanted replacement of the breeding material. Considering the number of reads per run, this technique offers an alternate method compared to others based on Sanger sequencing. Results gained using the HTS based approach are also supported by gene bank data gained using Sanger technology, which showed 100% identity (see unmarked accession numbers in Table S2).

To amplify all possible alleles might be rather challenging since the proximity of a recombination hotspot may keep resulting in high variance of amplicons in this region. 

Amplicon sequencing might be a solution for this issue, since sequencing noise is detectable as sequences of low coverage in comparison to Sanger sequencing. The advantage of amplicon sequencing is that these low abundance sequences can be filtered in silico, especially in cases where the number of the expected alleles is known.

Nevertheless, a more optimal and uniform priming efficiency targeting the hypervariable locus of the *csd* gene and decreasing the chances of generation of non-specific PCR products may allow reducing the coverage, making sequencing of more individuals possible while also lowering costs, which would especially benefit cases of sequencing the queens.

In Supplementary Table S1, we present the number of discarded alleles and their abundance. Supplementary Figure S2 shows PCR products visualized for Hq and Hd samples demonstrating their amplification success.

### 4.2. High Throughput Determination of csd Alleles from Honey Might Open the Way for Large-Scale Screening of Breeding Stocks But Has Limitations

Bee DNA extracted from honey samples enables a fast and efficient screening of *csd* alleles from a whole nucleus breeding stock of a mating yard. However, honey is an unfavourable environment for nucleic acids, as they are exposed to multiple forces, leading to their fragmentation in the long term. The approach presented here targets a small sequence of the *csd* gene and proved to be successful, as we managed to amplify useful DNA templates for high throughput sequencing in the case of eight honey samples.

At the current state, tracking alleles of the *csd* gene from samples of an unknown number of alleles implies technical ambiguities, since unspecific PCR products yielding irrelevant sequences may not make selecting the relevant sequences possible, especially in the case of the least abundant alleles. However, in amplicon filtering in the case of known alleles (e.g., diploid individual (worker or queen)), where non-specific products could be eliminated by setting the threshold at the two most abundant alleles, the situation is more challenging when the number of the expected alleles is unknown due to the peculiarities of the sample, as in the case of the honey. Unspecific amplicons might be eliminated if primers capable of more ideal amplification of the hypervariable region of the *csd* gene could be designed. However, this may not hold much promise, as new alleles might emerge due to the vicinity of a recombination hotspot.

Additional experiments would be needed to decide whether the *csd* allele composition of a honey sample alone may be sufficient for tracking its origin or if supplementation with markers on different loci would be necessary. More detailed genetic data from honey—*csd* alleles fortified with genotypes from other markers located in different loci, if necessary—may provide a molecular tool to determine its geographic origin as part of a profiling panel.

### 4.3. Tracking of csd Alleles Offers Potential Benefits for Closed Breeding Programmes Based on Instrumental Insemination

The heterozygosity of *csd* locus is influenced by anthropogenic impacts, such as hybridization of honey bees, provoked by international exchange of breeding materials (e.g., queen bees, bee sperm). Honey bees typically strive to avoid inbreeding by visiting drone congregation areas (DCA), which can contain drones from up to 240 colonies [33]. As this option is not available for those queens that are used in honey bee selection programs, other means are necessary to avoid inbreeding. Nonetheless, population studies based on *csd* sequence data are notable tools for the avoidance of diploid males in bee selection programs by allele-assisted breeding [34]. Balancing selection at the *csd* gene helps to avoid homozygosity in selected honey bee lines [16,34,35,36].

This is not the first study to analyse the HVR region of honey bee *csd* gene. Beye, et al. [37] found a high recombination rate in the sex determination region, whereas Liu, et al. [38] and Hasselmann and Beye [35] observed a high peak of recombination in the immediate vicinity of the sex determination locus. Hence, new alleles may appear continuously, as the HVR region is subject to a high level of recombination. As a result of such high levels of polymorphism, it is not surprising that the length and the exact position of the targeted HVR region show rather high levels of variability across these studies [7,9,11,18,21]. In order to retain the ability to compare all honey bee HVR sequences published thus far, we decided to target with our primers those conserved regions that were present in all sequences from the previous studies. Although this led to some loss of information from previously published alleles, this allowed us to compare our sequences to all previously determined *csd* alleles.

The introduction of HTS-based *csd* genotyping opens up the possibility of rapid generation of a large number of *csd* alleles within the next few years. As the current nomenclature is primarily based on alleles determined either by Sanger sequencing or HRM-based approaches that are clearly different in their sensitivity, we expect that old alleles will “pop up” again in HTS-based studies. We recommend that each research program and breeding facility should consider setting up their own labelling system and link their alleles to those determined earlier with the above technologies, if possible, in order to keep the possibility of cross-referencing open.

In case of proper timing, this method in practice may offer a possibility to gain *csd* allele data less labour-intensively in the case of the queens compared to traditional methods that inevitably implement molecular cloning in cases where there is need to gain sequence based data. As for a queen newly hatched, having about 7–13 days until insemination, the amount of time to complete the entire process from DNA extraction until sequencing takes less time by skipping molecular cloning and adjacent extensive Sanger sequencing of the clones if the queen rearing is scheduled according to HTS sequencing service. Nevertheless, it is restricted to cases when it is critical to be aware of the *csd* alleles because of the value of the breeding line. In most cases, to uncover the *csd* alleles out of the season, the process can be extended or postponed to establish an insemination plan for the next year, ensuring avoidance of the emergence of diploid drones.

## Figures and Tables

**Table 1:** Origin of the samples.

Country	Origin	Sample Type	Sample Code *
China	wild collected	bee, worker	Cw1
China	wild collected	bee, worker	Cw2
China	wild collected	bee, worker	Cw3.1 ^a^
China	wild collected	bee, worker	Cw3.2 ^a^
Hungary	private apiary, hive no 445/23	bee, worker	Hw1.1 ^b^
Hungary	private apiary, hive no 445/23	bee, worker	Hw1.2 ^b^
Hungary	private apiary, hive no 445/23	bee, drone	Hd1.1 ^c^
Hungary	private apiary, hive no 445/23	bee, drone	Hd1.2 ^c^
Hungary	private apiary, hive no 445/23	bee, drone	Hd1.3 ^c^
Hungary	private apiary (Kolics apiary)	bee, drone	Hd2
Hungary	private apiary (Kolics apiary)	bee, drone	Hd3.1 ^d^
Hungary	private apiary (Kolics apiary)	bee, drone	Hd3.2 ^d^
Hungary	private apiary (Kolics apiary)	bee, drone	Hd4
Hungary	private apiary (Kolics apiary)	bee, queen	Hq1.1 ^e^
Hungary	private apiary (Kolics apiary)	bee, queen	Hq1.2 ^e^
Hungary	private apiary (Kolics apiary)	bee, queen	Hq2.1 ^f^
Hungary	private apiary (Kolics apiary)	bee, queen	Hq2.2 ^f^
Hungary	private apiary (Kolics apiary)	bee, queen	Hq3.1 ^g^
Hungary	private apiary (Kolics apiary)	bee, queen	Hq3.2 ^g^
Hungary	private apiary (Kolics apiary)	bee, queen	Hq4
China	local grocery	honey	Lao
China	local grocery	honey	Chi
Georgia	private apiary	honey	Gru
Japan	private apiary	honey	Jap
Hungary	private apiary	honey	Gel
Hungary	private apiary	honey	Szg
Hungary	private apiary	honey	Ves
Hungary	private apiary, hive no 445/23	honey	445/23

*^, a, b, c, d, e, f, g^ Sample codes with the same letter in superscript are technical iterations of the same sample.

**Table 2:** Amino acid sequences of *csd* sex alleles isolated bee samples can be tracked in pedigree.

Sample Codes *	Amino Acid Sequence of *csd* Alleles	Coverage Used **	Coveragein in Total ***	Abundance of the Relevant Alleles
Cw1	IISSLSNKTIHNNNNYKYNYNNNNYNNNYNNNCKKLYYNIINI	4627	7848	71.34%
	IISSLSNKTIHNNNNYKYNYNNNYNNNNNYNNYNNTNYKKLYYNINYI	972		
Cw2	IISSLSNNYNYNNNNYNNYNNNYNKKLYYNINYI	4286	9280	80.23%
	IISSLSNNYNYSNYNNYNNNNYNNYKKLYYNINYI	3159		
Cw3.1 ^a^	IISSLSNNYNYSNYNNYNNYNNNYNNYKKLYYNINYI	11,699	23,013	78.38%
	IISSLSNKTIHNNNNYKYNYNNNNNNYKNYNNYKKLYYNINYI	6339		
Cw3.2 ^a^	IISSLSNKTIHNNNNYKYNYNNNNNNYKNYNNYKKLYYNINYI	7080	13,908	76.15%
	IISSLSNNYNYSNYNNYNNYNNNYNNYKKLYYNINYI	3511		
Hw1.1 ^b^	IISSLSNKTIHNNNNYKYNYNNNCKKLYYNINYI	15,779	31,092	81.03%
	**IISSLSNNYKYSNYNNYNNNNYNNNYNHYNNNYSKKLYYNINYI**	9416		
Hw1.2 ^b^	IISSLSNKTIHNNNNYKYNYNNNCKKLYYNINYI	13,505	30,441	81.30%
	**IISSLSNNYKYSNYNNYNNNNYNNNYNHYNNNYSKKLYYNINYI**	11,245		
Hd1.1 ^c^	**IISSLSNNYKYSNYNNYNNNNYNNNYNHYNNNYSKKLYYNINYI**	914	1325	68.98%
	-			
Hd1.2 ^c^	**IISSLSNNYKYSNYNNYNNNNYNNNYNHYNNNYSKKLYYNINYI**	17,489	19,280	90.71%
	-			
Hd1.3 ^c^	**IISSLSNNYKYSNYNNYNNNNYNNNYNHYNNNYSKKLYYNINYI**	139,098	149,456	93.1%
	-			
Hd2	IISSLSNNTIHNNNYKYNYNNNYNNYKKLYYNINYI	4074	4300	94.74%
	-			
Hd3.1 ^d^	IISSLSNNTIHNNNYKYNYNNNYNNYKKLYYNINYI	5431	5767	94.17%
	-			
Hd3.2 ^d^	IISSLSNNTIHNNNYKYNYNNNYNNYKKLYYNINYI	11,949	12,708	94.03%
	-			
Hd4	IISSLSNNTIHNNNYKYNYNNNYNNYKKLYYNINYI	14,886	15,985	93.12%
	-			
Hq1.1 ^e^	IISSLSNKTIHNNNNYNNNNNNYNNYNNYKKLYYNVINI	8701	19,005	86.35%
	IISSLSNNYKYSNYNNYNNNYNNYNNNYNNNYKKLYYNINYI	7710		
Hq1.2 ^e^	IISSLSNKTIHNNNNYNNNNNNYNNYNNYKKLYYNVINI	8056	19,625	80.63%
	IISSLSNNYKYSNYNNYNNNYNNYNNNYNNNYKKLYYNINYI	7767		
Hq2.1 ^f^	IISSLSNKTIHNNNNYNNNNYNNYKKLYYNIINI	14,248	20,762	86.76%
	IISSLSNNYNSNNYNNYNKYNYNNSKKLYYNINYI	3765		
Hq2.2 ^f^	IISSLSNKTIHNNNNYNNNNYNNYKKLYYNIINI	10,176	13,493	91.28%
	IISSLSNNYNSNNYNNYNKYNYNNSKKLYYNINYI	2140		
Hq3.1 ^g^	IISSLSNKTIHNNNNYNNNNNNYNNYNNYKKLYYNVINI	7130	14,775	88.90%
	IISSLSNNYKYSNYNNYNNNYNNYNNNYNNNYKKLYYNINYI	6005		
Hq3.2 ^g^	IISSLSNNYKYSNYNNYNNNYNNYNNNYNNNYKKLYYNINYI	6437	14,346	89.50%
	IISSLSNKTIHNNNNYNNNNNNYNNYNNYKKLYYNVINI	6402		
Hq4	IISSLSNKTIHNNNNYKPYYNINYI	11,434	22,417	91.19%
	IISSLSNNRNSNNYNNYNYKKLYYNINYI	9007		

* For detailed information on the sample codes, see Table 1. ** Coverage includes only the two most abundant sequences, that were considered to be relevant *** Coverage, involving also singleton sequences Coverage of the certain alleles are regarded as those sharing the same amino acid sequence at the hypervariable region. Upper indexes: same individuals in different iterations. Bold: one of the two alleles of the queen of hive 445/23 isolated from her drone and found in its female offspring. ^a, b, c, d, e, f, g^ Sample codes with the same letter in superscript are technical iterations of the same sample.

**Table 3:** New alleles reported in the present study.

Sample	Subspecies	Amino Acid Sequence of the Hypervariable Region	Total Coverage *	Abundance in the Sample	NCBI Accession Number
Cw1_SA2, Jap_SA12	*ligustica*	IISSLSNKTIHNNNNYKYNYNNNYNNNNNYNNYNNTNYKKLYYNINYI	4577 (972 + 3605)	12.4%, 1.5%	MK241931.1 identity to the most homologous existing sequence: 88%
Ves_SA4	*carnica*	IISSLSNKTIHDNNNYKYNYNNNNNNYKNYNNYKKLYYNINYI	1448	0.6%	MK241934.1 identity to the most homologous existing sequence: 98%
Ves_SA5	*carnica*	IISSLSNNYNYSNYNNYNNYNKNYNNYKKLYYNINYI	1317	0.5%	MK241935.1 identity to the most homologous existing sequence: 97%
Ves_SA6, Gru_SA4	*carnica/caucasica*	IISSLSNKTIHNNNNYKYNYNNNNNYKNYNNYKKLYYNINYI	2090 (1035 + 1055)	0.4%, 0,4%	MK241936.1 identity to the most homologous existing sequence: 98%
Ves_SA7, Chi_SA3, Gru_SA3	*carnica/caucasica*	IISSLSNKTIHNNNNYKYNYNNNNNYYKNYNNYKKLYYNINYI	3482 (1027 + 1383 + 1072)	0.4%, 0.7%, 0.4%	MK241937.1 identity to the most homologous existing sequence: 98%
SzG_SA4	*carnica*	IISSLSNKTIHNNNNYKYNYNNNNYNNNNYKKLQYYNINYI	22,572	3.5%	MK241933.1 identity to the most homologous existing sequence: 93%
Jap_SA14	*ligustica*	IISSLSNKTIHNNNNYNNNNYNNYNNNYNNNNYNNYKKLYYNINYI	1345	0.5%	MK241932.1 identity to the most homologous existing sequence: 96%

* Coverage includes only the two most abundant sequences, that were considered to be relevant.

## Supplementary Materials

The following are available online at https://www.mdpi.com/2075-4450/11/8/483/s1, Table S1. The number and abundance of discarded alleles. Table S2. Complete list of amino acid sequences of csd alleles isolated from honey bees and honeys with their occurrence. Figure S1. Aligned amino acid sequences of the hypervariable region of the csd gene. Figure S2. The gel electrophoresis image of the fragments.
