# Supplementary material for: An Alternative, High Throughput Method to Identify Csd Alleles of the Honey Bee
Source: Insects. 2020 Jul 30;11(8):483. doi: 10.3390/insects11080483 (PMC7469139; doi:10.3390/insects11080483)
Supplement: Supplementary file 1 [file insects-11-00483-s001.zip › Figure 1.docx]

Figure 1. Comparison of the two methods shows that traditional methods provides alternative only when drones are available with a compromise of more DNA extraction and PCR. However, if queen is directly targeted, it leads to extensive molecular cloning and sequencing. The advantage of the novel approach is disputable when investigating bulk samples.

*in order to successfully catch both alleles, 6 sequencing is optimal

**bee**

**Traditional method**

*Indirect determination of CSD alleles of the queen through her drones (if present)*

*-sample collection*

*-PCR reaction*

*-Sanger sequencing*

**Traditional method**

*Direct determination of CSD alleles of the queen us*

*-sample collection*

*-PCR reaction*

*-cloning into plasmid*

*-colony PCR reaction*

*-Sanger sequencing*

**Method described here**

*Direct determination of CSD alleles*

*of the queen*

*-sample collection from the wing*

*-PCR reaction*

*-preparation of the library for NGS*

PCR

COLONY

PCR

…ATGCTAC…

reads = 6*

reads = 1 000 to 15 000

PCR

*Library*

**A T C G T T G C A T G C T**

**T G T G T A C G T G C G T**

**T G C A A G T G**

**T G C A A C G T G**

**T G C A G T G C A G T G**

**T G C A G T G C T G**

**T G C A**

**T G C A G A G T G C**

**G C A A G T G C A T G C A**

**C A T C A T C A T T T C A**

+

PCR

…ATGCTAC…

reads = 6*

Genotyping of breeding queens – comparison of traditional methods with the one presented in this study

**Honey**

**Traditional method**

*Indirect determination of CSD alleles from the honey harvested from hives of a mating yard*

*-sample collection*

*-PCR reaction*

*-cloning into plasmid*

*-colony PCR reaction*

*-Sanger sequencing*

**Method described here**

*Indirect determination of CSD alleles*

*from the honey harvested from hives of a mating yard*

*- sample collection*

*-PCR reaction*

*-preparation of the library for NGS*

PCR

COLONY

PCR

…ATGCTAC…

reads = depending on

how many alleles are

aimed to catch

reads = 1 000 to 15 000

PCR

*Library*

**A T C G T T G C A T G C T**

**T G T G T A C G T G C G T**

**T G C A A G T G**

**T G C A A C G T G**

**T G C A G T G C A G T G**

**T G C A G T G C T G**

**T G C A**

**T G C A G A G T G C**

**G C A A G T G C A T G C A**

**C A T C A T C A T T T C A**

+

**HONEY**

harvested from

hives of the

mating yard

*CSD* alleles present in a breeding stock
