# Supplementary material for: An Alternative, High Throughput Method to Identify Csd Alleles of the Honey Bee
Source: Insects. 2020 Jul 30;11(8):483. doi: 10.3390/insects11080483 (PMC7469139; doi:10.3390/insects11080483)
Supplement: Supplementary file 1 [file insects-11-00483-s001.zip › Figure S1.docx]

5’

3’

*csd* gene

HVR

HVR F

HVR R


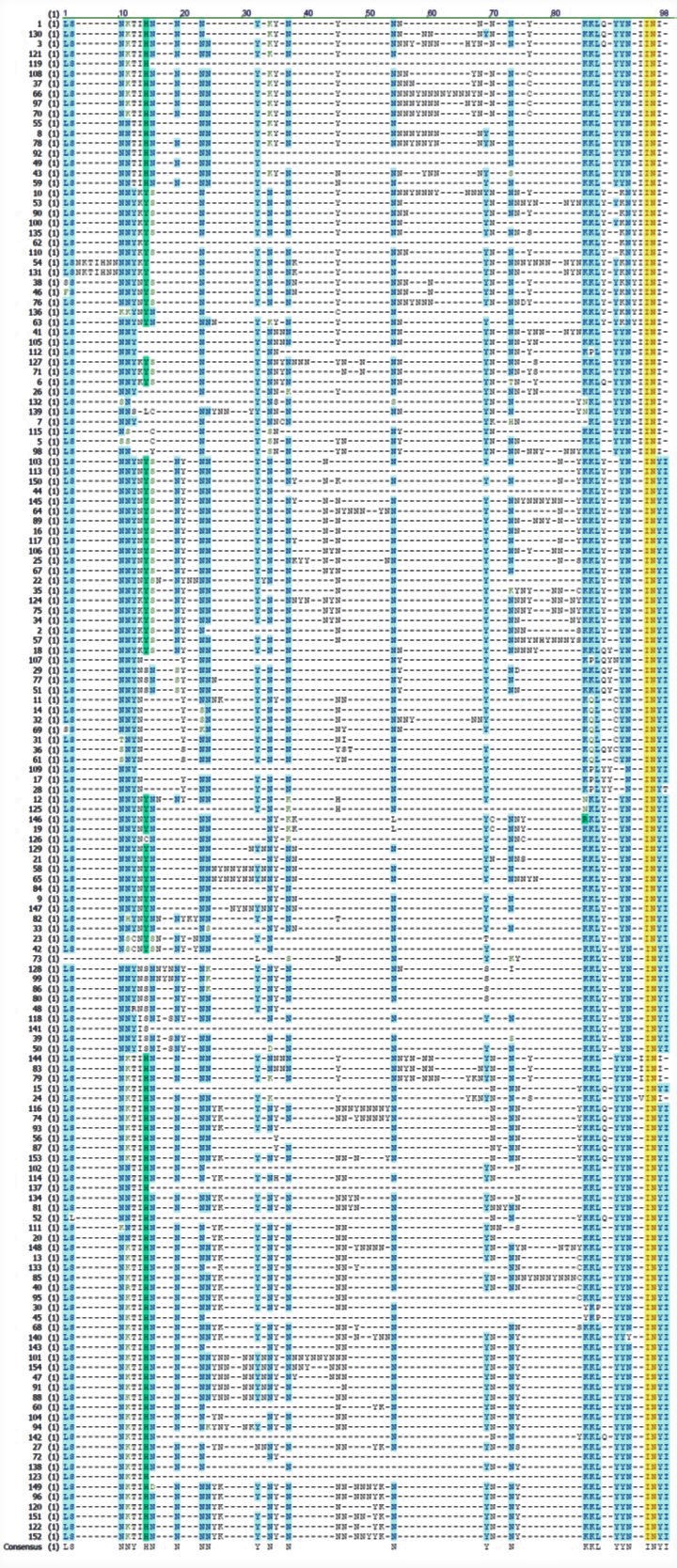

Figure S1. Aligned amino acid sequences of the hypervariable region of the *csd* gene.
