# Supplementary material for: An Alternative, High Throughput Method to Identify Csd Alleles of the Honey Bee
Source: Insects. 2020 Jul 30;11(8):483. doi: 10.3390/insects11080483 (PMC7469139; doi:10.3390/insects11080483)
Supplement: Supplementary file 1 [file insects-11-00483-s001.zip › Table S1.docx]

**Table S1.** The number and abundance of discarded alleles.

| **Sample codes*** | **Copy number total** | **Copy number used** | **Abundance of the relevant alleles** | | **Abundance of discarded sequences** | **Copy number of discarded sequences** | **Number of discarded alleles** | **Copy number of the most abundant discarded alleles** |
| --- | --- | --- | --- | --- | --- | --- | --- | --- |
| Hq1.1^e^ | 19 005 | 8 701 | 45.78% | 86.35% | 13.65% | 2 594 | 68 | 229 |
|  |  | 7 710 | 40.57% |  |  |  |  |  |
| Hq1.2^e^ | 19 625 | 8 056 | 41.05% | 80.63% | 19.37% | 3 802 | 65 | 516 |
|  |  | 7 767 | 39.58% |  |  |  |  |  |
| Hq2.1^f^ | 20 762 | 14 248 | 68.63% | 86.76% | 13.24% | 2 749 | 62 | 494 |
|  |  | 3 765 | 18.13% |  |  |  |  |  |
| Hq2.2^f^ | 13 493 | 10 176 | 75.42% | 91.28% | 8.72% | 1 177 | 33 | 176 |
|  |  | 2 140 | 15.86% |  |  |  |  |  |
| Hq3.1^g^ | 14 775 | 7 130 | 48.26% | 88.90% | 11.10% | 1 640 | 44 | 191 |
|  |  | 6 005 | 40.64% |  |  |  |  |  |
| Hq3.2^g^ | 14346 | 6 437 | 44.87% | 89.50% | 10.50% | 1507 | 41 | 175 |
|  |  | 6 402 | 44.63% |  |  |  |  |  |
| Hq4 | 22 417 | 11 434 | 51.01% | 91.19% | 8.81% | 1 976 | 37 | 361 |
|  |  | 9 007 | 40.18% |  |  |  |  |  |
| Hd2 | 4 300 | 4 074 | 94.74% | | 5.26% | 226 | 9 | 84 |
| Hd3.1^d^ | 5 767 | 5 431 | 94.17% | | 5.83% | 336 | 7 | 114 |
| Hd3.2^d^ | 12 708 | 11 949 | 94.03% | | 5.97% | 759 | 14 | 241 |
| Hd4 | 15 985 | 14 886 | 93.12% | | 6.88% | 1 099 | 28 | 297 |

Copy numbers of the certain alleles are regarded as those sharing the same amino acid sequence at the hypervariable region. *Upper indexes: same individuals in different iterations.
