# Supplementary material for: An Alternative, High Throughput Method to Identify Csd Alleles of the Honey Bee
Source: Insects. 2020 Jul 30;11(8):483. doi: 10.3390/insects11080483 (PMC7469139; doi:10.3390/insects11080483)
Supplement: Supplementary file 1 [file insects-11-00483-s001.zip › Table S2.docx]

Table S2. Complete list of amino acid sequences of *csd* alleles isolated from honey bees and honeys with their occurrence

| **Sample** | **Sample** |  |  | **Copy** | **Abundance** | **worker bee** | **worker bee** | **queen** | **drone** | **honey** | **honey** | **honey** | **honey** | **honey** | **honey** | **honey** |  |
| --- | --- | --- | --- | --- | --- | --- | --- | --- | --- | --- | --- | --- | --- | --- | --- | --- | --- |
| **type** | **code** | **subspecies** | **amino acid sequence of the hypervariable region** | **number*** |  | **Cw** | **Hw 1.1** | **Hq** | **Hd** | **445.23** | **Ves** | **Szg** | **Gel** | **Chi** | **Gru** | **Jap** | **NCBI** |
| worker bee | Cw1_SA1 | unknown | IISSLSNKTIHNNNNYKYNYNNNNYNNNYNNNCKKLYYNIINI | 4 627 | 59.0% | / | - | - | - | - | - | - | - | - | - | Jap_SA8 | AEI99762.1 |
| worker bee | Cw1_SA2 | unknown | IISSLSNKTIHNNNNYKYNYNNNYNNNNNYNNYNNTNYKKLYYNINYI + | 972 | 12.4% | / | - | - | - | - | - | - | - | - | - | Jap_SA12 | MK241931.1 |
| worker bee | Cw2_SA1 | unknown | IISSLSNNYNYNNNNYNNYNNNYNKKLYYNINYI | 4 286 | 46.2% | / | - | - | - | - | - | - | - | - | - | Jap_SA11 | CCF23487.1 |
| worker bee | Cw2_SA2 | unknown | IISSLSNNYNYSNYNNYNNNNYNNYKKLYYNINYI | 3 159 | 34.0% | / | - | - | - | - | - | - | - | - | - | Jap_SA10 | CCF23496.1 |
| worker bee | Cw3_SA1 | unknown | IISSLSNNYNYSNYNNYNNYNNNYNNYKKLYYNINYI | 11 699 | 50.8% | / | - | - | - | 445/23_SA1 | Ves_SA1 | - | Gel_SA1 | Chi_SA1 | Gru_SA1 | Jap_SA1 | CCF23508.1 |
| worker bee | Cw3_SA2 | unknown | IISSLSNKTIHNNNNYKYNYNNNNNNYKNYNNYKKLYYNINYI | 6 339 | 27.5% | / | - | - | - | 445/23_SA2 | Ves_SA2 | - | Gel_SA2 | Chi_SA2 | Gru_SA2 | Jap_SA3 | AGA84535.1 |
|  |  |  |  |  |  |  |  |  |  |  |  |  |  |  |  |  |  |
| worker bee | **Hw1.1&1.2_SA1** | carnica | IISSLSNKTIHNNNNYKYNYNNNCKKLYYNINYI | 13 505 & 15 779 | 44.4% & 50.7% | - | / | - | - | 445/23_SA4 | - | - | Gel_SA5 | - | - | Jap_SA4 | ABD14096.1 |
| worker bee | **Hw1.1&1.2_SA2** | carnica | **IISSLSNNYKYSNYNNYNNNNYNNNYNHYNNNYSKKLYYNINYI** | **11 245 & 9 146** | **36.9% & 30.3%** | - | / | - | Hd1.1&1.2&_SA*2* | 445/23_SA3 | Ves_SA3 | - | Gel_SA3 | - | - | Jap_SA7 | AQZ41225.1 |
|  |  |  |  |  |  |  |  |  |  |  |  |  |  |  |  |  |  |
| queen | Hq1.1&1.2_SA1 | carnica | IISSLSNKTIHNNNNYNNNNNNYNNYNNYKKLYYNVINI | 8 701 & 8 056 | 45.78% & 41.01% | - | - | Hq3.1&3.2_SA1 | - | - | - | - | - | - | - | - | [QEN96053.1](https://www.ncbi.nlm.nih.gov/protein/QEN96053.1?report=genbank&log$=prottop&blast_rank=1&RID=BUBT7T50014) |
| queen | Hq1.1&1.2_SA2 | carnica | IISSLSNNYKYSNYNNYNNNYNNYNNNYNNNYKKLYYNINYI | 7 710 & 7 767 | 40.57% & 39.58% | - | - | Hq3.1&3.2_SA2 | - | 445/23_SA5 | - | - | - | - | - | - | CCF23533.1 |
| queen | Hq2.1&2.2_SA1 | carnica | IISSLSNKTIHNNNNYNNNNYNNYKKLYYNIINI | 14 248 & 10 176 | 68.63% & 75.42% | - | - | / | - | - | - | - | - | - | - | - | [AEI99777.1](https://www.ncbi.nlm.nih.gov/protein/AEI99777.1?report=genbank&log$=prottop&blast_rank=1&RID=BUC5KT8V014) |
| queen | Hq2.1&2.2_SA2 | carnica | IISSLSNNYNSNNYNNYNKYNYNNSKKLYYNINYI | 3 765 & 2 104 | 18.13% & 15.86% | - | - | / | - | - | - | - | - | - | - | - | [QEN96022.1](https://www.ncbi.nlm.nih.gov/protein/QEN96022.1?report=genbank&log$=prottop&blast_rank=1&RID=BUCB2HAU014) |
| queen | Hq3.1&3.2_SA1 | carnica | IISSLSNKTIHNNNNYNNNNNNYNNYNNYKKLYYNVINI | 7 130 & 6 402 | 48.26% & 44.63% | - | - | Hq1.1&1.2_SA1 | - | - | - | - | - | - | - | - | [QEN96053.1](https://www.ncbi.nlm.nih.gov/protein/QEN96053.1?report=genbank&log$=prottop&blast_rank=1&RID=BUBT7T50014) |
| queen | Hq3.1&3.2_SA2 | carnica | IISSLSNNYKYSNYNNYNNNYNNYNNNYNNNYKKLYYNINYI | 6 005 & 6 437 | 40.64% & 44.87% | - | - | Hq1.1&1.2_SA2 | - | 445/23_SA5 | - | - | - | - | - | - | CCF23533.1 |
| queen | Hq4_SA1 | carnica | IISSLSNKTIHNNNNYKPYYNINYI | 11 434 | 51.01% | - | - | / | - | - | - | - | - | - | - | - | [AGA84534.1](https://www.ncbi.nlm.nih.gov/protein/AGA84534.1?report=genbank&log$=prottop&blast_rank=1&RID=BUCWYXE5016) |
| queen | Hq4_SA2 | carnica | IISSLSNNRNSNNYNNYNYKKLYYNINYI | 9 007 | 40.18% | - | - | / | - | - | - | - | - | - | - | - | [CCF23473.1](https://www.ncbi.nlm.nih.gov/protein/CCF23473.1?report=genbank&log$=prottop&blast_rank=1&RID=BUD067BC016) |
|  |  |  |  |  |  |  |  |  |  |  |  |  |  |  |  |  |  |
| drone | **Hd1.1&1.2_SA*2&*** | carnica | **IISSLSNNYKYSNYNNYNNNNYNNNYNHYNNNYSKKLYYNINYI** | **17 489 & 914** | **90.71% & 68.98%** | - | Hw1_SA2 | - | / | 445/23_SA3 | Ves_SA3 | - | Gel_SA3 | - | - | Jap_SA7 | AQZ41225.1 |
|  | **Hd1.3** |  |  | **139 098** | **93.1%** |  |  |  |  |  |  |  |  |  |  |  |  |
| drone | Hd2_SA1 | carnica | IISSLSNNTIHNNNYKYNYNNNYNNYKKLYYNINYI | 4 074 | 94.74% | - | - | - | Hd3&4_SA1 | - | - | SzG_SA6 | - | - | - | - | ART88483.1 |
| drone | Hd3.1&3.2_SA1 | carnica | IISSLSNNTIHNNNYKYNYNNNYNNYKKLYYNINYI | 5 431 & 11 949 | 94.17% & 94.03% | - | - | - | Hd2&4_SA1 | - | - | SzG_SA6 | - | - | - | - | ART88483.1 |
| drone | Hd4_SA1 | carnica | IISSLSNNTIHNNNYKYNYNNNYNNYKKLYYNINYI | 14 886 | 93.12% | - | - | - | Hd2&3_SA1 | - | - | SzG_SA6 | - | - | - | - | ART88483.1 |
|  |  |  |  |  |  |  |  |  |  |  |  |  |  |  |  |  |  |
| honey | 445/23_SA1 | carnica | IISSLSNNYNYSNYNNYNNYNNNYNNYKKLYYNINYI | 154 100 | 62.3% | Cw3_SA1 | - | - | - | / | Ves_SA1 | - | Gel_SA1 | Chi_SA1 | Gru_SA1 | Jap_SA1 | CCF23508.1 |
| honey | 445/23 _SA2 | carnica | IISSLSNKTIHNNNNYKYNYNNNNNNYKNYNNYKKLYYNINYI | 38 583 | 15.6% | Cw3_SA2 | - | - | - | / | Ves_SA2 | - | Gel_SA2 | Chi_SA2 | Gru_SA2 | Jap_SA3 | AGA84535.1 |
| **honey** | **445/23_SA3** | carnica | **IISSLSNNYKYSNYNNYNNNNYNNNYNHYNNNYSKKLYYNINYI** | 27 834 | 11.3% | - | *Hw1_SA2* | - | Hd1.1&1.2_SA*2* | / | Ves_SA3 | - | Gel_SA3 | - | - | Jap_SA7 | AQZ41225.1 |
| **honey** | **445/23_SA4** | carnica | IISSLSNKTIHNNNNYKYNYNNNCKKLYYNINYI | 4 595 | 1.9% | - | Hw1.1_SA1 | - | - | / | - | - | Gel_SA5 | - | - | Jap_SA4 | ABD14096.1 |
| honey | 445/23_SA5 | carnica | IISSLSNNYKYSNYNNYNNNYNNYNNNYNNNYKKLYYNINYI | 2 451 | 1.0% | - | - | Hq1&3_SA2 | - | / | - | - | - | - | - | - | CCF23533.1 |
|  |  |  |  |  |  |  |  |  |  |  |  |  |  |  |  |  |  |
| honey | Ves_SA1 | carnica | IISSLSNNYNYSNYNNYNNYNNNYNNYKKLYYNINYI | 132 543 | 50.9% | Cw3_SA1 | - | - | - | 445/23_SA1 | / | - | Gel_SA1 | Chi_SA1 | Gru_SA1 | Jap_SA1 | CCF23508.1 |
| honey | Ves_SA2 | carnica | IISSLSNKTIHNNNNYKYNYNNNNNNYKNYNNYKKLYYNINYI | 75 578 | 29.0% | Cw3_SA2 | - | - | - | 445/23_SA2 | / | - | Gel_SA2 | Chi_SA2 | Gru_SA2 | Jap_SA3 | AGA84535.1 |
| honey | Ves_SA3 | carnica | **IISSLSNNYKYSNYNNYNNNNYNNNYNHYNNNYSKKLYYNINYI** | 27 774 | 10.7% | - | ***Hw1.1_SA2*** | - | - | 445/23_SA3 | / | - | Gel_SA3 | - | - | Jap_SA7 | AQZ41225.1 |
| honey | Ves_SA4 | carnica | IISSLSNKTIHDNNNYKYNYNNNNNNYKNYNNYKKLYYNINYI + | 1 448 | 0.6% | - | - | - | - | - | / | - | - | - | - | - | MK241934.1 |
| honey | Ves_SA5 | carnica | IISSLSNNYNYSNYNNYNNYNKNYNNYKKLYYNINYI + | 1 317 | 0.5% | - | - | - | - | - | / | - | - | - | - | - | MK241935.1 |
| honey | Ves_SA6 | carnica | IISSLSNKTIHNNNNYKYNYNNNNNYKNYNNYKKLYYNINYI + | 1 035 | 0.4% | - | - | - | - | - | / | - | - | - | Gru_SA4 | - | MK241936.1 |
| honey | Ves_SA7 | carnica | IISSLSNKTIHNNNNYKYNYNNNNNYYKNYNNYKKLYYNINYI + | 1 027 | 0.4% | - | - | - | - | - | / | - | - | Chi_SA3 | Gru_SA3 | - | MK241937.1 |
|  |  |  |  |  |  |  |  |  |  |  |  |  |  |  |  |  |  |
| honey | SzG_SA1 | carnica | IISSLSNKTIHNNNNYNNYKKLYYNINYI | 371 153 | 57.2% | - | - | - | - | - | - | / | - | - | - | - | CCF23474.1 |
| honey | SzG_SA2 | carnica | IISSLSNKTIHNNNNYKYNYNNNNYNNNCKKLYYNIINI | 220 185 | 33.9% | - | - | - | - | - | - | / | - | - | - | - | ART88599.1 92/100 |
| honey | SzG_SA3 | carnica | IISSLSNKTIHNNNKYNYNKYNYNNNNYNNYKKLYYNINYI | 27 128 | 4.2% | - | - | - | - | - | - | / | - | - | - | - | CCF23526.1 |
| honey | SzG_SA4 | carnica | IISSLSNKTIHNNNNYKYNYNNNNYNNNNYKKLQYYNINYI + | 22 572 | 3.5% | - | - | - | - | - | - | / | - | - | - | - | MK241933.1 |
| honey | SzG_SA5 | carnica | IISSLSNNYNYSNYNNYNNNYNNYNKKLYYNINYI | 3 758 | 0.6% | - | - | - | - | - | - | / | - | - | - | - | ABD14117.1 |
| honey | SzG_SA6 | carnica | IISSLSNNTIHNNNYKYNYNNNYNNYKKLYYNINYI | 3 127 | 0.5% | - | - | - | Hd2&3&4_SA1 | - | - | / | - | - | - | - | ART88483.1 88/100 |
| honey | SzG_SA7 | carnica | IISSLSNKTIHNNNNYKYNYNNNYNNNCKKLYYNIINI | 741 | 0.1% | - | - | - | - | - | - | / | - | - | - | - | AGA84536.1 |
|  |  |  |  |  |  |  |  |  |  |  |  |  |  |  |  |  |  |
| honey | Gel_SA1 | unknown | IISSLSNNYNYSNYNNYNNYNNNYNNYKKLYYNINYI | 120786 | 49.3% | Cw3_SA1 | - | - | - | 445/23_SA1 | Ves_SA1 | - | / | Chi_SA1 | Gru_SA1 | Jap_SA1 | CCF23508.1 |
| honey | Gel_SA2 | unknown unknown | IISSLSNKTIHNNNNYKYNYNNNNNNYKNYNNYKKLYYNINYI | 70775 | 28.9% | Cw3_SA2 | - | - | - | 445/23_SA2 | Ves_SA2 | - | / | Chi_SA2 | Gru_SA2 | Jap_SA3 | AGA84535.1 |
| honey | Gel_SA3 | unknown | **IISSLSNNYKYSNYNNYNNNNYNNNYNHYNNNYSKKLYYNINYI** | 17940 | 7.3% | - | Hw1.1_SA2 | - | - | 445/23_SA3 | Ves_SA3 | - | / | - | - | Jap_SA7 | AQZ41225.1 |
| honey | Gel_SA4 | unknown | IISSLSNNYKYSNYNNYNNYNKKLYYKNYIINI | 8821 | 3.6% | - | - | - | - | - | - | - | / | - | - | Jap_SA6 | AGA84523.1 |
| honey | Gel_SA5 | unknown | IISSLSNKTIHNNNNYKYNYNNNCKKLYYNINYI | 1436 | 0.6% | - | Hw1.1_SA1 | - | - | 445/23_SA4 | - | - | / | - | - | Jap_SA4 | ABD14096.1 |
|  |  |  |  |  |  |  |  |  |  |  |  |  |  |  |  |  |  |
| honey | Chi_SA1 | unknown | IISSLSNNYNYSNYNNYNNYNNNYNNYKKLYYNINYI | 134899 | 67.8% | Cw3_SA1 | - | - | - | 445/23_SA1 | Ves_SA1 | - | Gel_SA1 | / | Gru_SA1 | Jap_SA1 | CCF23508.1 |
| honey | Chi_SA2 | unknown | IISSLSNKTIHNNNNYKYNYNNNNNNYKNYNNYKKLYYNINYI | 47725 | 24.0% | Cw3_SA2 | - | - | - | 445/23_SA2 | Ves_SA2 | - | Gel_SA2 | / | Gru_SA2 | Jap_SA3 | AGA84535.1 |
| honey | Chi_SA3 | unknown | IISSLSNKTIHNNNNYKYNYNNNNNYYKNYNNYKKLYYNINYI + | 1383 | 0.7% | - | - | - | - | - | Ves_SA7 | - | - | / | Gru_SA3 | - | MK241937.1 |
|  |  |  |  |  |  |  |  |  |  |  |  |  |  |  |  |  |  |
| honey | Gru_SA1 | caucasica | IISSLSNNYNYSNYNNYNNYNNNYNNYKKLYYNINYI | 151190 | 61.0% | Cw3_SA1 | - | - | - | 445/23_SA1 | Ves_SA1 | - | Gel_SA1 | Chi_SA1 | / | Jap_SA1 | CCF23508.1 |
| honey | Gru_SA2 | caucasica | IISSLSNKTIHNNNNYKYNYNNNNNNYKNYNNYKKLYYNINYI | 75049 | 30.3% | Cw3_SA2 | - | - | - | 445/23_SA2 | Ves_SA2 | - | Gel_SA2 | Chi_SA2 | / | Jap_SA3 | AGA84535.1 |
| honey | Gru_SA3 | caucasica | IISSLSNKTIHNNNNYKYNYNNNNNYYKNYNNYKKLYYNINYI | 1072 | 0.4% | - | - | - | - | - | Ves_SA7 | - | - | Chi_SA3 | / | - | MK241937.1 |
| honey | Gru_SA4 | caucasica | IISSLSNKTIHNNNNYKYNYNNNNNYKNYNNYKKLYYNINYI | 1055 | 0.4% | - | - | - | - | - | Ves_SA6 | - | - | - | / | - | MK241936.1 |
|  |  |  |  |  |  |  |  |  |  |  |  |  |  |  |  |  |  |
| honey | Jap_SA1 | ligustica | IISSLSNNYNYSNYNNYNNYNNNYNNYKKLYYNINYI | 43115 | 17.4% | Cw3_SA1 | - | - | - | 445/23_SA1 | Ves_SA1 | - | Gel_SA1 | Chi_SA1 | Gru_SA1 | / | CCF23508.1 |
| honey | Jap_SA2d | ligustica | IISSLSNNYNYNNNYNNYNNNYNKKLYYNINYI | 29235 | 11.8% | - | - | - | - | - | - | - | - | - | - | / | ART88551.1 87/100 |
| honey | Jap_SA3 | ligustica | IISSLSNKTIHNNNNYKYNYNNNNNNYKNYNNYKKLYYNINYI | 25733 | 10.4% | Cw3_SA2 | - | - | - | 445/23_SA2 | Ves_SA2 | - | Gel_SA2 | Chi_SA2 | Gru_SA2 | / | AGA84535.1 |
| honey | Jap_SA4 | ligustica | IISSLSNKTIHNNNNYKYNYNNNCKKLYYNINYI | 27565 | 11.1% | - | Hw1.1_SA1 | - | - | 445/23_SA4 | - | - | Gel_SA5 | - | - | / | ABD14096.1 |
| honey | Jap_SA5d | ligustica | IISSLSNKTIHNNNNYKNYNNYKNYNNYKNYNNYKKLYYNINYI | 38554 | 15.5% | - | - | - | - | - | - | - | - | - | - | / | ART88538.1 90/100 |
| honey | Jap_SA6 | ligustica | IISSLSNNYKYSNYNNYNNYNKKLYYKNYIINI | 15493 | 6.2% | - | - | - | - | - | - | - | Gel_SA4 | - | - | / | AGA84523.1 |
| honey | Jap_SA7 | ligustica | **IISSLSNNYKYSNYNNYNNNNYNNNYNHYNNNYSKKLYYNINYI** | 16553 | 6.7% | - | Hw1.1_SA2 | - | - | 445/23_SA3 | Ves_SA3 | - | Gel_SA3 | - | - | / | AQZ41225.1 |
| honey | Jap_SA8 | ligustica | IISSLSNKTIHNNNNYKYNYNNNNYNNNYNNNCKKLYYNIINI | 9875 | 4.0% | Cw1_SA1 | - | - | - | - | - | - | - | - | - | / | AEI99762.1 |
| honey | Jap_SA9 | ligustica | IISSLSNNYNYSNYNNNNYKQLCYNINYI | 4187 | 1.7% | - | - | - | - | - | - | - | - | - | - | / | AGA84531.1 |
| honey | Jap_SA10 | ligustica | IISSLSNNYNYSNYNNYNNNNYNNYKKLYYNINYI | *3342* | *1.3%* | Cw2_SA2 | - | - | - | - | - | - | - | - | - | / | CCF23496.1 |
| honey | Jap_SA11 | ligustica | IISSLSNNYNYNNNNYNNYNNNYNKKLYYNINYI | 3691 | 1.5% | Cw2_SA1 | - | - | - | - | - | - | - | - | - | / | CCF23487.1 |
| honey | Jap_SA12 | ligustica | IISSLSNKTIHNNNNYKYNYNNNYNNNNNYNNYNNTNYKKLYYNINYI | *3605* | *1.5%* | Cw1_SA2 | - | - | - | - | - | - | - | - | - | / | MK241931.1 |
| honey | Jap_SA13 | ligustica | IISSLSNNYKYSNYNNYNNNYNNYNNNYKKLYYNINYI | *842* | *0.3%* | - | - | - | - | - | - | - | - | - | - | / | AEI99787.1 |
| honey | Jap_SA14 | ligustica | IISSLSNKTIHNNNNYNNNNYNNYNNNYNNNNYNNYKKLYYNINYI + | *1345* | *0.5%* | - | - | - | - | - | - | - | - | - | - | / | MK241932.1 |

Copy numbers of the certain alleles are regarded as those sharing the same amino acid sequence at the hypervariable region.

Legend: *Bold*: Scattering of the allele of queen of hive 445/23 isolated from her drone offspring.

* Copy number includes only the most abundant sequences, that were considered to be relevant

*+/in colour*: Novel alleles. Considered as those that differ in the amino acid order compared to deposited gene bank sequences.

*Sequences with accession number marked yellow*: Sequences that shares sequence identity of the given gene bank sequence, but do differ in the coverage. Regarded as potentially novel allele.
