# Supplementary material for: An Alternative, High Throughput Method to Identify Csd Alleles of the Honey Bee
Source: Insects. 2020 Jul 30;11(8):483. doi: 10.3390/insects11080483 (PMC7469139; doi:10.3390/insects11080483)
Supplement: Supplementary file 1 [file insects-11-00483-s001.zip › Table1.docx]

**Table 1.** Origin of the samples.

| **Country** | **Origin** | **Sample type** | **Sample code*** |
| --- | --- | --- | --- |
| China | local grocery | honey | Lao |
| China | local grocery | honey | Chi |
| Georgia | private apiary | honey | Gru |
| Japan | private apiary | honey | Jap |
| Hungary | private apiary | honey | Gel |
| Hungary | private apiary | honey | Szg |
| Hungary | private apiary | honey | Ves |
| Hungary | private apiary, hive no 445/23 | honey | 445/23 |
| China | wild collected | bee, worker | Cw1 |
| China | wild collected | bee, worker | Cw2 |
| China | wild collected | bee, worker | Cw3.1^a^ |
| China | wild collected | bee, worker | Cw3.2^a^ |
| Hungary | private apiary, hive no 445/23 | bee, worker | Hw1.1^b^ |
| Hungary | private apiary, hive no 445/23 | bee, worker | Hw1.2^b^ |
| Hungary | private apiary, hive no 445/23 | bee, drone | Hd1.1^c^ |
| Hungary | private apiary, hive no 445/23 | bee, drone | Hd1.2^c^ |
| Hungary | private apiary, hive no 445/23 | bee, drone | Hd1.3^c^ |
| Hungary | private apiary (Kolics apiary) | bee, drone | Hd2 |
| Hungary | private apiary (Kolics apiary) | bee, drone | Hd3.1^d^ |
| Hungary | private apiary (Kolics apiary) | bee, drone | Hd3.2^d^ |
| Hungary | private apiary (Kolics apiary) | bee, drone | Hd4 |
| Hungary | private apiary (Kolics apiary) | bee, queen | Hq1.1^e^ |
| Hungary | private apiary (Kolics apiary) | bee, queen | Hq1.2^e^ |
| Hungary | private apiary (Kolics apiary) | bee, queen | Hq2.1^f^ |
| Hungary | private apiary (Kolics apiary) | bee, queen | Hq2.2^f^ |
| Hungary | private apiary (Kolics apiary) | bee, queen | Hq3.1^g^ |
| Hungary | private apiary (Kolics apiary) | bee, queen | Hq3.2^g^ |
| Hungary | private apiary (Kolics apiary) | bee, queen | Hq4 |

* Sample codes with the same letter in superscript are technical iterations of the same sample.
