# Supplementary material for: An Alternative, High Throughput Method to Identify Csd Alleles of the Honey Bee
Source: Insects. 2020 Jul 30;11(8):483. doi: 10.3390/insects11080483 (PMC7469139; doi:10.3390/insects11080483)
Supplement: Supplementary file 1 [file insects-11-00483-s001.zip › Table2 rev.docx]

**Table 2.** Amino acid sequences of *csd* alleles isolated bee samples can be tracked in pedigree

| Sample codes* | Amino acid sequence of *csd* alleles | Coverage used** | Coverage in total*** | Abundance of the relevant alleles |
| --- | --- | --- | --- | --- |
| Cw1 | IISSLSNKTIHNNNNYKYNYNNNNYNNNYNNNCKKLYYNIINI | 4 627 | 7 848 | 71.34% |
|  | IISSLSNKTIHNNNNYKYNYNNNYNNNNNYNNYNNTNYKKLYYNINYI | 972 |  |  |
| Cw2 | IISSLSNNYNYNNNNYNNYNNNYNKKLYYNINYI | 4 286 | 9 280 | 80.23% |
|  | IISSLSNNYNYSNYNNYNNNNYNNYKKLYYNINYI | 3 159 |  |  |
| Cw3.1^a^ | IISSLSNNYNYSNYNNYNNYNNNYNNYKKLYYNINYI | 11 699 | 23 013 | 78.38% |
|  | IISSLSNKTIHNNNNYKYNYNNNNNNYKNYNNYKKLYYNINYI | 6 339 |  |  |
| Cw3.2^a^ | IISSLSNKTIHNNNNYKYNYNNNNNNYKNYNNYKKLYYNINYI | 7 080 | 13 908 | 76.15% |
|  | IISSLSNNYNYSNYNNYNNYNNNYNNYKKLYYNINYI | 3 511 |  |  |
| Hw1.1^b^ | IISSLSNKTIHNNNNYKYNYNNNCKKLYYNINYI | 15 779 | 31 092 | 81.03% |
|  | **IISSLSNNYKYSNYNNYNNNNYNNNYNHYNNNYSKKLYYNINYI** | 9 416 |  |  |
| Hw1.2^b^ | IISSLSNKTIHNNNNYKYNYNNNCKKLYYNINYI | 13 505 | 30 441 | 81.30% |
|  | **IISSLSNNYKYSNYNNYNNNNYNNNYNHYNNNYSKKLYYNINYI** | 11 245 |  |  |
| Hd1.1^c^ | **IISSLSNNYKYSNYNNYNNNNYNNNYNHYNNNYSKKLYYNINYI** | 914 | 1 325 | 68.98% |
|  | - |  |  |  |
| Hd1.2^c^ | **IISSLSNNYKYSNYNNYNNNNYNNNYNHYNNNYSKKLYYNINYI** | 17 489 | 19 280 | 90.71% |
|  | - |  |  |  |
| Hd1.3 ^c^ | **IISSLSNNYKYSNYNNYNNNNYNNNYNHYNNNYSKKLYYNINYI** | 139 098 | 149 456 | 93.1% |
|  |  |  |  |  |
| Hd2 | IISSLSNNTIHNNNYKYNYNNNYNNYKKLYYNINYI | 4 074 | 4 300 | 94.74% |
|  | - |  |  |  |
| Hd3.1^d^ | IISSLSNNTIHNNNYKYNYNNNYNNYKKLYYNINYI | 5 431 | 5 767 | 94.17% |
|  | - |  |  |  |
| Hd3.2^d^ | IISSLSNNTIHNNNYKYNYNNNYNNYKKLYYNINYI | 11 949 | 12 708 | 94.03% |
|  | - |  |  |  |
| Hd4 | IISSLSNNTIHNNNYKYNYNNNYNNYKKLYYNINYI | 14 886 | 15 985 | 93.12% |
|  | - |  |  |  |
| Hq1.1^e^ | IISSLSNKTIHNNNNYNNNNNNYNNYNNYKKLYYNVINI | 8 701 | 19 005 | 86.35% |
|  | IISSLSNNYKYSNYNNYNNNYNNYNNNYNNNYKKLYYNINYI | 7 710 |  |  |
| Hq1.2^e^ | IISSLSNKTIHNNNNYNNNNNNYNNYNNYKKLYYNVINI | 8 056 | 19 625 | 80.63% |
|  | IISSLSNNYKYSNYNNYNNNYNNYNNNYNNNYKKLYYNINYI | 7 767 |  |  |
| Hq2.1^f^ | IISSLSNKTIHNNNNYNNNNYNNYKKLYYNIINI | 14 248 | 20 762 | 86.76% |
|  | IISSLSNNYNSNNYNNYNKYNYNNSKKLYYNINYI | 3 765 |  |  |
| Hq2.2^f^ | IISSLSNKTIHNNNNYNNNNYNNYKKLYYNIINI | 10 176 | 13 493 | 91.28% |
|  | IISSLSNNYNSNNYNNYNKYNYNNSKKLYYNINYI | 2 140 |  |  |
| Hq3.1^g^ | IISSLSNKTIHNNNNYNNNNNNYNNYNNYKKLYYNVINI | 7 130 | 14 775 | 88.90% |
|  | IISSLSNNYKYSNYNNYNNNYNNYNNNYNNNYKKLYYNINYI | 6 005 |  |  |
| Hq3.2^g^ | IISSLSNNYKYSNYNNYNNNYNNYNNNYNNNYKKLYYNINYI | 6 437 | 14 346 | 89.50% |
|  | IISSLSNKTIHNNNNYNNNNNNYNNYNNYKKLYYNVINI | 6 402 |  |  |
| Hq4 | IISSLSNKTIHNNNNYKPYYNINYI | 11 434 | 22 417 | 91.19% |
|  | IISSLSNNRNSNNYNNYNYKKLYYNINYI | 9 007 |  |  |

* For detailed information on the sample codes, see Table 1.

** Coverage includes only the two most abundant sequences, that were considered to be relevant

*** Coverage, involving also singleton sequences

Coverage of the certain alleles are regarded as those sharing the same amino acid sequence at the hypervariable region. Upper indexes: same individuals in different iterations. Bold: one of the two alleles of the queen of hive 445/23 isolated from her drone and found in its female offspring.
