# Supplementary material for: An Alternative, High Throughput Method to Identify Csd Alleles of the Honey Bee
Source: Insects. 2020 Jul 30;11(8):483. doi: 10.3390/insects11080483 (PMC7469139; doi:10.3390/insects11080483)
Supplement: Supplementary file 1 [file insects-11-00483-s001.zip › Table3 rev.docx]

| **Sample type** | **Sample** | **Subspecies** | **Amino acid sequence of the hypervariable region** | **Total coverage*** | **Abundance in the**  **sample** | **NCBI accession number** |
| --- | --- | --- | --- | --- | --- | --- |
| worker bee | Cw1_SA2, Jap_SA12 | *ligustica* | IISSLSNKTIHNNNNYKYNYNNNYNNNNNYNNYNNTNYKKLYYNINYI | 4 577 (972+3 605) | 12.4%, 1.5% | MK241931.1 identity to the most homologous existing sequence: 88% |
| honey | Ves_SA4 | *carnica* | IISSLSNKTIHDNNNYKYNYNNNNNNYKNYNNYKKLYYNINYI | 1 448 | 0.6% | MK241934.1 identity to the most homologous existing sequence: 98% |
| honey | Ves_SA5 | *carnica* | IISSLSNNYNYSNYNNYNNYNKNYNNYKKLYYNINYI | 1 317 | 0.5% | MK241935.1 identity to the most homologous existing sequence: 97% |
| honey | Ves_SA6, Gru_SA4 | *carnica / caucasica* | IISSLSNKTIHNNNNYKYNYNNNNNYKNYNNYKKLYYNINYI | 2 090 (1 035+1 055) | 0.4%, 0,4% | MK241936.1 identity to the most homologous existing sequence: 98% |
| honey | Ves_SA7, Chi_SA3, Gru_SA3 | *carnica / caucasica* | IISSLSNKTIHNNNNYKYNYNNNNNYYKNYNNYKKLYYNINYI | 3 482 (1 027+1 383+1 072) | 0.4%, 0.7%, 0.4% | MK241937.1 identity to the most homologous existing sequence: 98% |
| honey | SzG_SA4 | *carnica* | IISSLSNKTIHNNNNYKYNYNNNNYNNNNYKKLQYYNINYI | 22 572 | 3.5% | MK241933.1 identity to the most homologous existing sequence: 93% |
| honey | Jap_SA14 | *ligustica* | IISSLSNKTIHNNNNYNNNNYNNYNNNYNNNNYNNYKKLYYNINYI | 1 345 | 0.5% | MK241932.1 identity to the most homologous existing sequence: 96% |

**Table 3.** New alleles reported in the present study

* Coverage includes only the two most abundant sequences, that were considered to be relevant
