# Supplementary figures and images for: An Alternative, High Throughput Method to Identify Csd Alleles of the Honey Bee
Source: Insects. 2020 Jul 30;11(8):483. doi: 10.3390/insects11080483 (PMC7469139; doi:10.3390/insects11080483)

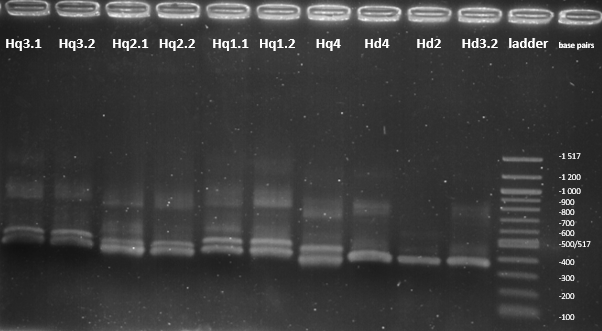


Figure S2. The gel electrophoresis image of the fragments.

Supplement: Supplementary file 1 [file insects-11-00483-s001.zip › Figure S2.docx]
